# Signature based on metabolic‐related gene pairs can predict overall survival of osteosarcoma patients

**DOI:** 10.1002/cam4.3984

**Published:** 2021-05-28

**Authors:** Long‐Qing Li, Liang‐Hao Zhang, Yao‐bo Yuan, Xin‐Chang Lu, Yi Zhang, Yong‐Kui Liu, Jia Wen, Manhas Abdul Khader, Tao Liu, Jia‐Zhen Li, Yan Zhang

**Affiliations:** ^1^ Department of Orthopaedic Surgery The First Affiliated Hospital of Zhengzhou University Zhengzhou Henan PR China; ^2^ Department of Urology The First Affiliated Hospital of Zhengzhou University Zhengzhou Henan PR China; ^3^ Department of Orthopedics Gushi County People’s Hospital Xinyang Henan PR China

**Keywords:** metabolic reprogramming, MRGP, osteosarcoma, prognosis, TCGA, tumour immunology

## Abstract

**Background:**

Osteosarcoma is a tumour of malignant origin in children and adolescents. Recent progression indicates that it is necessary to develop new therapies to improve the patient's prognosis rather than strengthen anti‐tumour chemotherapy. Researchers recently realised that cancer is a kind of disease with a metabolic disorder, and metabolic reprogramming is becoming a new cancer hallmark. Hence, our study's primary purpose is to explore the value of genes related to osteosarcoma metabolism.

**Methods:**

From public databases, three osteosarcoma datasets with adequate clinical information were obtained. Besides, the IMvigor dataset through the ‘IMvigor’ package as a supplement was downloaded, the metabolic‐related genes were identified, and these genes were used to construct the metabolic‐related gene pairs (MRGP). Based on the prognosis‐related MRGP, two molecular subtypes were identified. There are significant differences in the metabolic characteristics between the two molecular subtypes. Subsequently, the MRGP signature is constructed using the least absolute shrinkage and selection operator regression method. Finally, use SubMap analysis to evaluate the response of patients in the MRPG signature group to immunotherapy.

**Results:**

The MRGP signature can reliably predict overall survival in patients with osteosarcoma. The MRGP signature is also associated with osteosarcoma patients’ metastatic status and can be used for subsequent risk classification of metastatic patients. The immunotherapy is more likely to benefit the patients in the MRGP low‐risk group.

**Conclusion:**

Metabolic‐related gene pairs signature can assess the prognosis of patients with osteosarcoma.

## Graphical abstract

Metabolism‐related gene pair signatures can predict the overall survival of patients with osteosarcoma and are related to tumor metastasis in patients with osteosarcoma.Patients in the MRGP low‐risk group may benefit from immunotherapy

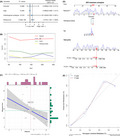

## 1 INTRODUCTION

Osteosarcoma is a rare tumour of malignant origin that primarily affects children and adolescents.^1^ Since the introduction of chemotherapy in 1970, the 5‐year survival rate of non‐metastatic osteosarcoma has reached 70%.^2^ Also, most patients can receive limb salvage surgery and obtain proper postoperative limb function.^3^, ^4^ Even though many efforts have been made, osteosarcoma patients’ therapeutic strategies and outcomes are still not ideal. Recent progression indicates that it is necessary to develop new therapies to improve the patient's prognosis rather than strengthen anti‐tumour chemotherapy.^5^, ^6^ Therefore, it is essential to evaluate the underlying molecular mechanism of osteosarcoma diversity.

Researchers have realised that cancer is a kind of disease with a metabolic disorder.^7^, ^8^ As early as 1920, Otto Warburg has observed that even in the presence of large amounts of oxygen, some tumour cells still preferentially rely on glycolysis.^9^ Now, with the development of research technology, essential changes in metabolic pathways in cancer have been discovered. For example, liver cancer with glutamate metabolism and prostate cancer with fatty acid metabolism have been reported.^10^, ^11^ Therefore, metabolic reprogramming is becoming a new hallmark of cancer.^12^ However, cancers from different sources express metabolic genes and pathways differently.^13^ The role and mechanism of these metabolic genes in osteosarcoma remain unclear.

More recently, by utilizing high‐throughput gene detection technology, whole‐genome analysis of mRNA expression profiles has been used to explore the complex biological behaviour of cancer and determine new tumour classification methods.^14^, ^15^ In this study, we constructed research that focused on metabolic gene pairs based on the associated ranking of the extent of metabolic gene expression and developed signatures based on metabolic gene pairs to improve risk stratification of osteosarcoma patients explored the relationship between signatures and metastasis.

## 2 METHODS AND MATERIALS

### 2.1 Collection of data

Our team collected the transcriptome data (FPKM) of 88 patients with osteosarcoma from the Cancer Genome Atlas database (TCGA, http://cancerge‐nome.nih.gov/) and the clinical data of the corresponding patients were obtained from the TARGET database. Two of the patients had no survival status, and one patient had no overall survival time, so these three patients were not included in further analysis. We then collected osteosarcoma patients’ available data from the Gene Expression Omnibus database (GEO, http://www.ncbi.nlm.nih.gov/geo/), patients without survival data were excluded. Simultaneously, the GSE21257 dataset (Based on the GPL10295 platform) and the GSE39055 dataset (Based on the GPL14951 platform) were included in our research. Among them, the GSE21257 dataset contains the overall survival and metastasis time of 53 patients with osteosarcoma, and the GSE39055 dataset contains the recurrence‐free survival of 37 patients with osteosarcoma. Recurrence‐free survival is defined as the time until the first recurrence or the latest follow‐up. One of the patients had a recurrence‐free survival of 0 and was not included in further analysis. Table 1 shows the details of the osteosarcoma datasets involved in this study. Moreover, with the help of R software's ‘IMvigor’ software package, we obtained the IMvigor data set, which contained clinical information and RNA‐seq data of patients who had metastatic urothelial cancer (mUC) treated with immune checkpoint inhibitors (PD‐L1).^16^ We used the IMvigor data set to study the signature‐immunotherapy efficiency relationship.

**TABLE 1:** Summary of clinical characteristics of osteosarcoma patient data sets in the study

Characteristic	(TCGA *n* = 88)	(GSE21257 *n* = 53)	(GSE39055 *n* = 36)
Vital status, *n* (%)			
Alive	57(64.8)	30(62.3)	26(72.2)
Dead	29(33.0)	23(37.7)	10(27.8)
Unknown	2(0.2)	0(0)	0(0)
Age, *n* (%)			
>=18	19(21.6)	20(37.7)	3(8.3)
<18	69(78.4)	33(62.3)	33(91.7)
Gender, *n* (%)			
Male	51(58.0)	34(64.2)	19(52.8)
Female	37(42.0)	19(35.8)	17(47.2)
Metastasis, *n* (%)			
M0	66(75)	19(35.8)	NA
M1	22(25)	34(64.2)	NA
Histological, *n* (%)			
Osteoblastic	NA	32(60.4)	NA
Other	NA	21(39.6)	NA
Recurrence, *n* (%)			
NO	NA	NA	18(50)
YES	NA	NA	18(50)

Abbreviation: NA, not available.

### 2.2 Identify prognostic‐related metabolic gene pairs

From the molecular signature database, we downloaded the KEGG pathway file ‘c2.cp.kegg.v7.0.symbols’ and used Perl to search for genes related to metabolic pathways. Specifically, use Perl to identify all metabolism‐related pathways and extract all the genes in those pathways. The metabolic genes present in all data sets were then identified for further analysis. Table S1 lists the identified metabolic related genes. Comparing the metabolically related genes in each sample was performed in pairs to calculate the metabolic‐related gene pair (MRGP). More specifically, in a particular MRGP, if the first metabolic gene's expression level is higher than the second metabolic gene, the MRGP value is 1; otherwise, it is 0 (For example, for the gene pair CYP2S1|SULT1A1, if a patient's CYP2S1 gene expression is higher than SULT1A1, then the value of the patient's gene pair CYP2S1|SULT1A1 is 1). If a particular MRGP has a score of 0 or 1 in more than 80% of the samples in any data set, the MRGP is excluded. We used Cox univariate regression analysis to evaluate the relationship between each MRGP and prognosis in the osteosarcoma cohort. MRGP with *p* < 0.05 was taken for further analysis. These MRGPs are listed in Table S2.

### 2.3 Identify MRGP‐based molecular subgroups in patients with osteosarcoma

First, based on the prognosis‐related MRGP, patients with osteosarcoma with overall survival information are divided into different molecular subgroups through consensus clustering (CC). Consensus clustering is an unsupervised clustering method. Our research performed consensus clustering through the ‘CancerSubtypes’ software package.^17^ Then through the log‐rank test, the Kaplan–Meier curve explores the difference in survival between the molecular subgroups. Moreover, the ‘limma’ software package of R software was used to identify differentially expressed genes (DEG) between molecular subgroups of the TCGA cohort.^18^ The gene possessing absolute log2 FC>1 and adjusted *p* < 0.01 is called DEG. We then used the ‘clusterProfiler’ package to do functional and pathway enrichment analysis on DEG, with the significance threshold set to adjusted *p* < 0.05.^19^

### 2.4 Build and evaluate MRGP signature

Lasso penalised Cox regression was applied to select the most important prognostic markers from the previously determined prognosis MRGP.^20^ First, we performed 1,000 iterations with the least absolute shrinkage and selection operator (LASSO). When a particular MRGP appeared more than 50 times in 1,000 iterations, the MRGP was considered a robust MRGP. Subsequently, these robust MRGPs are included in the proportional risk model, respectively, and we then calculated the area under the receiver's operating characteristic curve (AUROC).^21^ The optimal model is considered when AUROC attains peak level. Risk scores for all patients were calculated based on the optimal model, and the optimal threshold for risk scores was determined by analysis of the time‐dependent receiver operating characteristics curve (TROC).^22^ According to the cut‐off value, patients were divided into low‐risk groups and high‐risk groups, and the difference in survival rate between the two groups was calculated by log‐rank test. Furthermore, we performed a multivariate Cox analysis to determine whether the MRGP signature is independent of clinical characteristics. Finally, the prognostic values of MRGP signatures and clinical characteristics were compared by time‐dependent ROC analysis.

### 2.5 Estimation of immune infiltrates

First, we performed an immune infiltration assessment through the ‘microenvironment cell population count (MCP‐counter)’ method.^23^ The MCP‐counter method can evaluate the absolute abundance of 8 immune cells and 2 stromal cell populations through transcriptome data. Recent studies have shown that the immune cell infiltration score inferred by the MCP‐counter method can be used for comparison between samples and performs well.^24^ Due to the requirements of the MCP‐counter method, log2 conversion is performed on the FPKM data of TARGET‐OS, and the converted data is used as an input file. We also calculated the immune score, matrix score, and estimated score through the ‘ESTIMATE’ software package.^25^

### 2.6 Analysis of gene set variation

Gene set variation analysis (GSVA) can be defined as a non‐parametric unsupervised gene set enrichment that assesses potential changes in pathway activity.^26^ GSVA analysis was carried out based on 114 metabolic gene characteristics and gene set files ‘c2.cp.kegg.v7.0.symbols’ using the ‘GSVA’ package.^13^ Subsequently, our team used the ‘limma’ package of the R software to compare the GSVA scores between patients in different groups. Signatures with log2 FC>0.2 and adjusted *p* < 0.05 were regarded as differentially expression signatures.

### 2.7 Construction and verification of MRGP nomogram

A nomogram based on the MRGP signature was built using the ‘regplot’ package. We used the concordance index (C‐index) and calibration chart to assess the nomogram's predictive ability and precision, respectively. Subsequently, the clinical utility of the nomogram was evaluated using a decision curve analysis (DCA). DCA was originally a method of evaluating the benefits of diagnostic tests, and recently it is often used to evaluate the net benefits of nomograms.^27^, ^28^

### 2.8 Predict the benefit of immunotherapy to patients with osteosarcoma

First, we use the tumour microenvironmental immune type (TMIT) to evaluate the efficacy of immunotherapy (anti‐PD‐L1).^29^, ^30^ TMIT classification divides patients into four clusters based on the expression of CD8A and CD274 genes. Recent pan‐cancer analysis results showed that TMTI classification could indirectly predict the response of tumour patients to immunotherapy. With the help of the SubMap Analysis (gene model), the transcriptome data of melanoma patients treated with immune checkpoint inhibitors and osteosarcoma patients were compared to indirectly predict osteosarcoma patients’ immunotherapy response.^31^, ^32^ Due to the requirements of SubMap analysis, we downloaded the log2 converted Count data of the TARGET‐OS dataset from UCSC Xena datasets as the input file.

### 2.9 Statistical analysis

All statistical analyses in this research were conducted using R software (version 3.6.3, R Foundation for Statistical Computing, Vienna, Austria). We used the Kolmogorov–Smirnov method to test the normality of variables. The unpaired Student's *t*‐test was used to evaluate the difference between two groups of variables normally distributed, and the Mann–Whitney *U*‐test was used to evaluate the difference between two groups of variables with non‐normally distributed. The correlation between the two variables is evaluated using Spearman correlation analysis. Analyse contingency table variables by Fisher's exact test. If no special instructions, *p* < 0.05 is considered statistically significant.

## 3 RESULTS

### 3.1 Characteristics of patients with osteosarcoma and identification of prognosis‐related MRGP

Table 1 provides information on all osteosarcoma patient data sets used in this study. As mentioned earlier, we first identified 824 metabolic‐related genes present in each data set used in this study. Use these 824 genes to construct metabolic‐related gene pairs (MRGP), and delete MRGP with relatively small changes (more than 80% of samples in any data set score 1 or 0), and the remaining MRGPs are used for further analysis. Subsequently, we identified 1118 prognostic‐related MRGPs in the cohort of patients with osteosarcoma by univariate cox regression analysis (*p* < 0.05). Table S2 shows the details of these MRGPs.

### 3.2 Molecular subgroups construction using prognostic related MRGP

**FIGURE 1:**
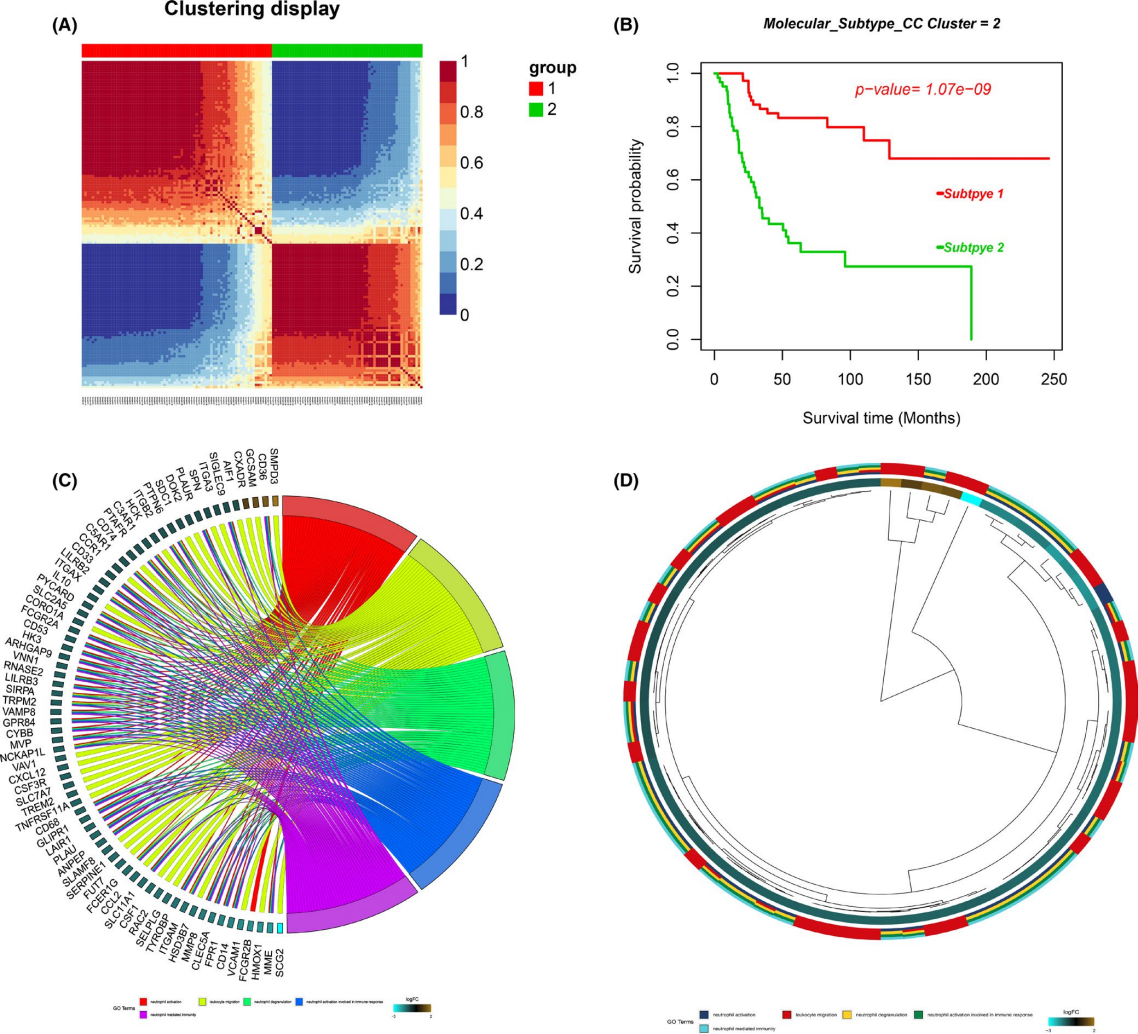
Consensus clustering of metabolism related gene pairs (MRGP) in osteosarcoma. (A) Consensus matrices of osteosarcoma patients for k = 2; (B) Based on the CC clustering of 1118 MRGPs, osteosarcoma patients are divided into two molecular subtypes, and the overall survival of the two groups of patients is significantly different; (C) Gene Ontology (GO) enrichment analysis of DEG. Chord plot displays the relationship between genes and GO terms. (D) Hierarchical clustering of the gene expression profiles in each GO term

First, with the help of the Consensus Clustering method, we divided 138 osteosarcoma patients with overall survival information (TARGET‐OS and GSE21257) into different molecular subgroups based on 1118 prognostic‐related MRGP. As shown in Figure 1A, two molecular subtypes were identified: metabolic cluster 1 (MC1) and metabolic cluster 2 (MC2). Kaplan–Meier curve results show that patients with the MC1 cluster have longer overall survival compared to the MC2 cluster (Figure 1B). We further focus on the TCGA data set, which has the most osteosarcoma patients and the most comprehensive mRNA expression data. To better characterise the two molecular subgroups, we conducted a differential expression analysis. Genes with absolute log2 FC>2 and adjusted *p*‐value < 0.05 are considered differentially expressed genes. In total, we identified 478 differentially expressed genes and Table S3 lists the information of these genes. Subsequently, Gene Ontology (GO) enrichment analysis of differentially expressed genes was conducted using the ‘clusterProfiler’ software package. Table S4 shows the detailed results of the enrichment analysis. As shown in Figure 1CD, the differentially expressed genes between the two clusters of patients are enriched in pathways such as neutrophil activation, leukocyte migration, positive regulation of cytokine production and T‐cell activation.

**FIGURE 2:**
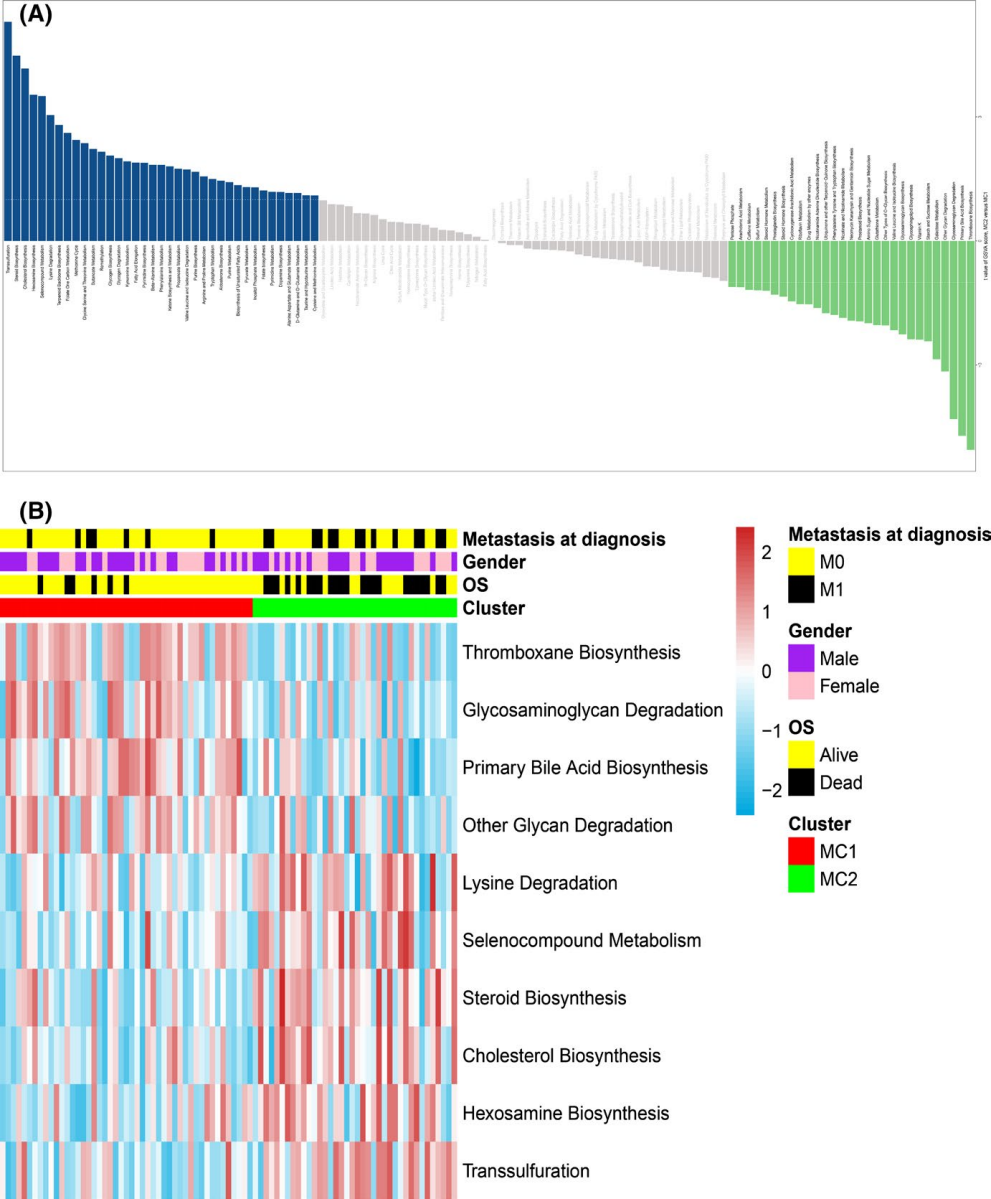
Associations between metabolism and the osteosarcoma molecular subtypes. (A) The bar plot shows the specific metabolism‐associated signatures of the two molecular subtypes. (B) Heatmap of metabolism‐associated signatures with significant differences between the two molecular subtypes. Metastasis status, overall survival status and gender (metastasis and died, black; no metastasis and alive, yellow; male, purple; female, pink) are indicated above the heatmap

Since our classification is based on metabolic related gene pairs, we further explored the differences in metabolic signatures among different molecular subtypes. As described in the method, we obtained 114 metabolic processes (Table S5) scores of TCGA patients using the ‘GSVA’ software package (Figure 2A). Table S6 shows the detailed results. Subsequently, differential expression analysis was conducted to explore the differences in the metabolic processes of the two molecular subtypes. As shown in Figure 2AB total of 10 differentially expression signatures were identified between the 2 clusters of patients. Among them, MC1 patients had higher scores for 4 metabolic processes, and MC2 patients had higher scores for the other 6 metabolic processes. Table S7 provides detailed results of differential expression analysis.

### 3.3 Construction of MRGP signature related to prognosis

**FIGURE 3:**
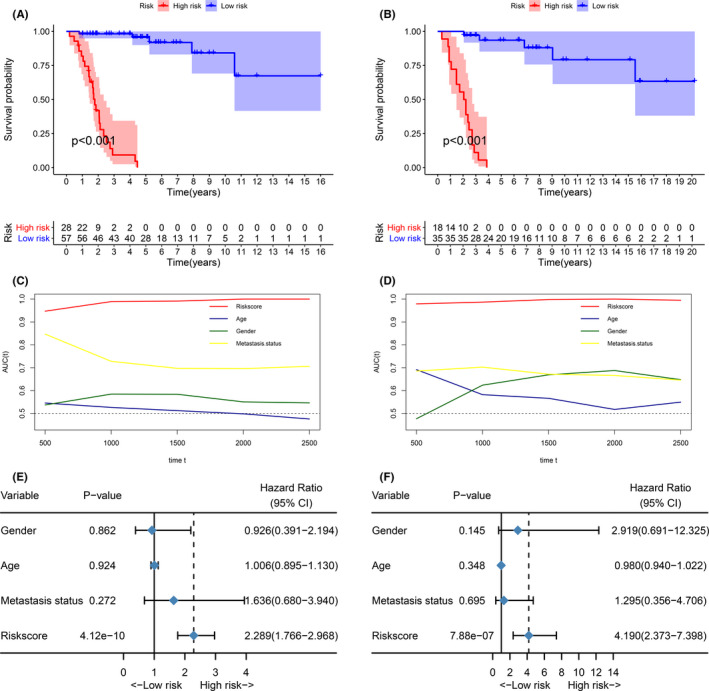
MRGP signatures are a good predictor of overall survival in patients with osteosarcoma. (A) Kaplan‐Meier survival curve of the overall survival of MRGP signature group patients in the TCGA cohort. (B) Kaplan‐Meier survival curve of the overall survival of MRGP signature group patients in the GSE21257 cohort. (C) Time‐dependent ROC curves of overall survival of MRGP signature group patients in the TCGA cohort. (D) Time‐dependent ROC curves of overall survival of MRGP signature group patients in the GSE21257 cohort. (E) Forest plots of the relationship between various variables and overall survival in the TCGA cohort. (F) Forest plots of the relationship between various variables and overall survival in the GSE21257 cohort

For establishing a clinically usable signature, important MRGP must be identified to reduce the dimension. We used the expression matrix of the prognosis‐related MRGP as an input file and performed 1,000 iterations with the help of the least absolute shrinkage and selection operator. A total of 55 MRGPs appeared more than 50 times in 1000 iterations. As described in the method, these genes are sequentially included in the proportional risk model, and we calculated the AUROC. The signature composed of 39 MRGPs was finally determined. Table S8 provides detailed information and coefficients for 39 MRPG. The optimal cut‐off of the MRGP signature for estimation of osteosarcoma's overall survival was identified to be 3.447 using TROC curve analysis. According to the cut‐off values, we divided patients into two groups. The results show that patients’ prognosis in the MRGP high‐risk group is poor (Figure S1). Besides separating TCGA and GSE21257 patients, patients with high MRGP signature scores still have a poor prognosis (Figure 3AB). Based on TROC results, it is clear that the MRGP score always has an excellent prognostic ability. With patients’ prolonged survival time, the prognostic value of metastatic state continues to decline (Figure 3CD). We further investigated whether the MRGP signature can be used as an independent predictor of overall survival in the GSE21257 and TCGA cohorts through multivariate Cox regression analysis. The results of multivariate Cox analysis, including clinical and pathological factors (age, gender, sarcoma subtype and metastatic status), show that the MRGP signature is an independent prognostic factor (Figure 3EF).

Subsequently, patients with overall survival information were divided into different subgroups according to age, gender and metastatic status. The predictive ability of MRGP signatures was tested in various subgroups. As shown in Figure S2, patients with high MRGP signature values in each subgroup have a poor prognosis.

**FIGURE 4:**
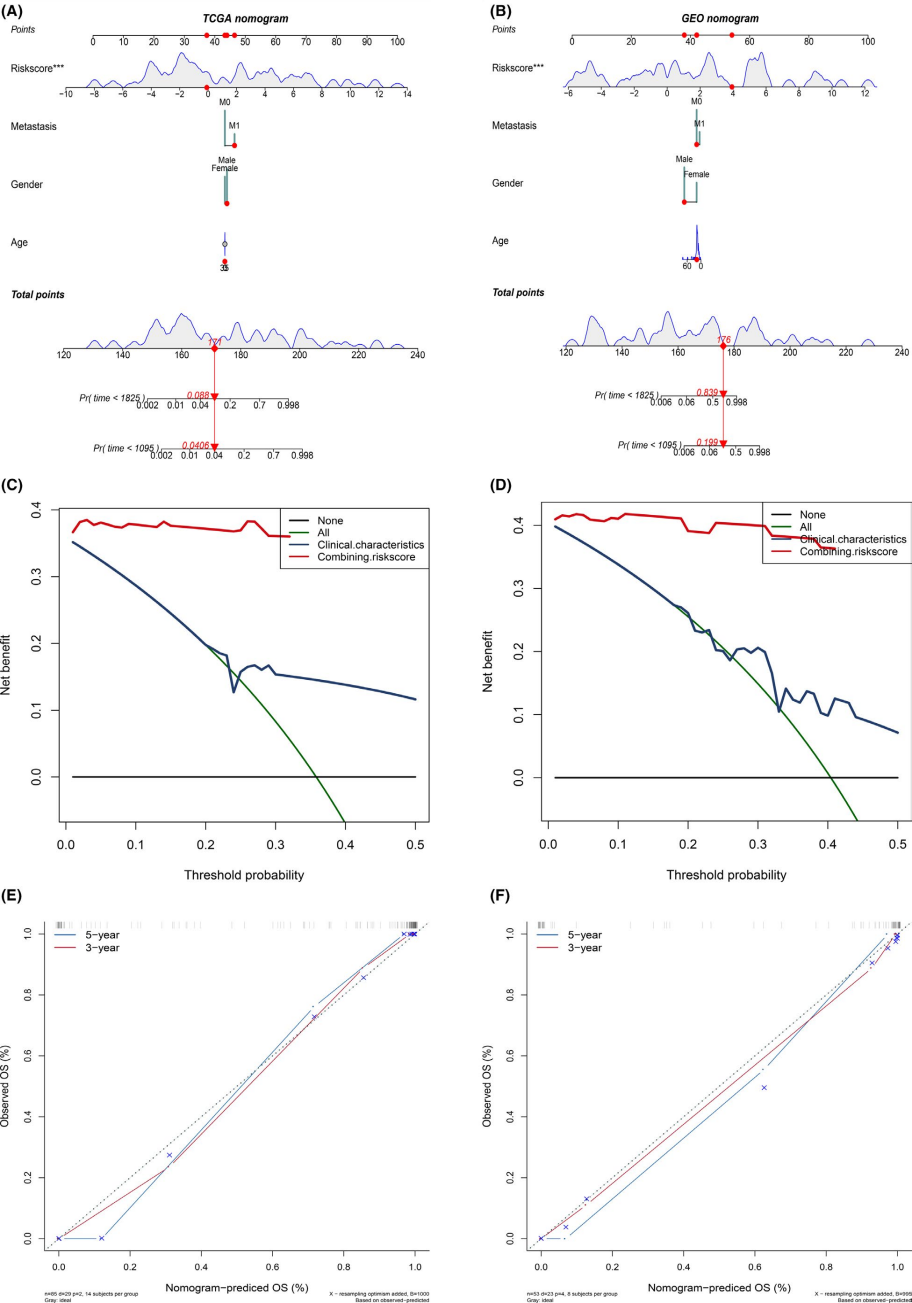
Build and verify nomogram based on MRGP. (A) A nomogram for predicting the prognosis of TCGA patients based on MRGP values and clinical variables; (B) A nomogram for predicting the prognosis of GSE21257 patients based on MRGP values and clinical variables; (C) Calibration chart in the TCGA cohort to verify the accuracy of the nomogram; (D) Calibration chart in the GSE21257 cohort to verify the accuracy of the nomogram. (E) Decision curve analysis of nomogram in TCGA cohort. (F) Decision curve analysis of nomogram in GSE21257 cohort

Finally, based on the MRGP signature, the prediction of osteosarcoma patients’ overall survival was determined by constructing a nomogram (Figure 4AB). As shown in Figure 4CD, the calibration curve shows that the MRGP nomogram can accurately predict the overall survival in patients with osteosarcoma. Also, the C‐index of the MRGP nomogram is 0.954 and 0.962, indicating that the MRGP nomogram has excellent discrimination. The results of DCA analysis show that combining MRGP signatures with clinical characteristics can bring net clinical benefits (Figure 4EF).

### 3.4 MRGP signature can predict patient's recurrence‐free survival

**FIGURE 5:**
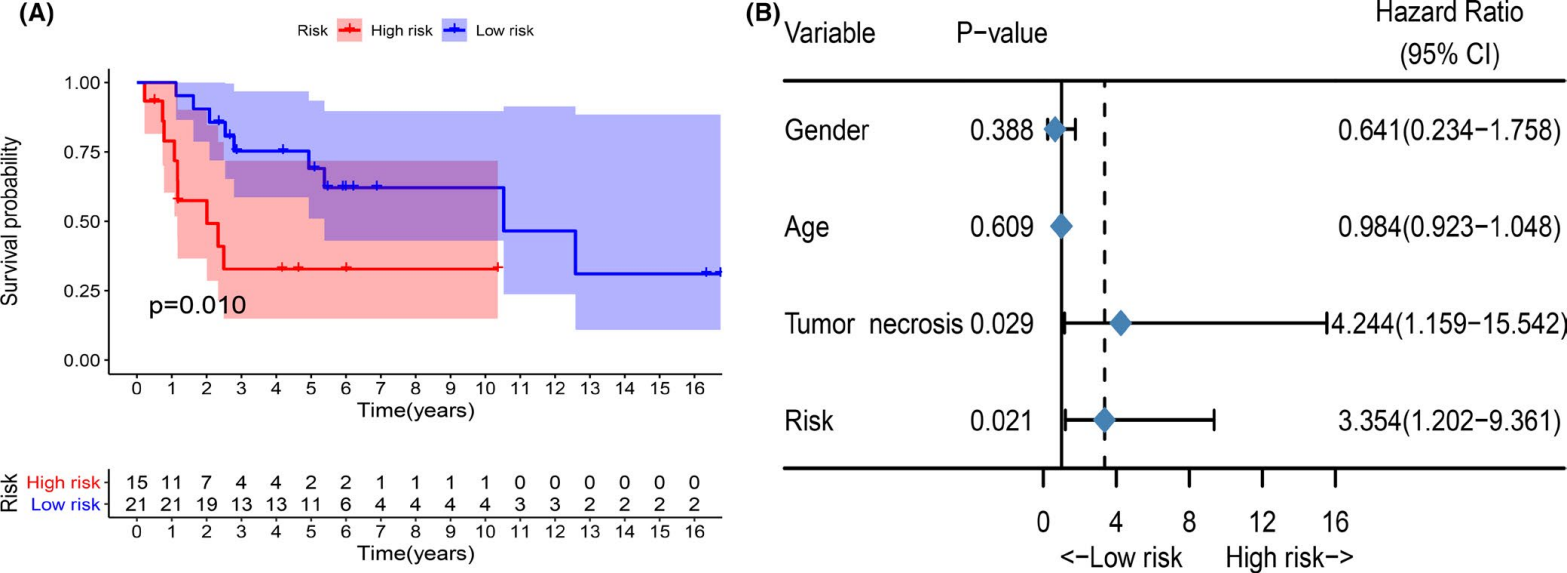
MRGP signature can predict recurrence‐free survival of patients with osteosarcoma. (A) Kaplan‐Meier survival curve of the recurrence‐free survival of MRGP signature group patients in the GSE39055 cohort. (B) Forest plots of the associations between various variables and recurrence‐free survival in the GSE39055 cohort

We included the GSE39055 data set in the dataset containing only recurrence‐free survival information for patients with osteosarcoma. Therefore, this data set does not participate in the construction of MRGP signatures. We used the GSE39055 data set as a verification set and used TROC analysis to determine the value of the MRGP signature to predict RFS in osteosarcoma patients. Similarly, the patients were classified into two groups based on the cut‐off value (6.760). As shown in Figure 5A, patients with high MRGP signatures have a higher risk of recurrence. Subsequently, a multivariate analysis was conducted, and the results showed that the MRGP signature was an independent risk factor (Figure 5B).

### 3.5 MRGP signature predicts tumour metastasis

**FIGURE 6:**
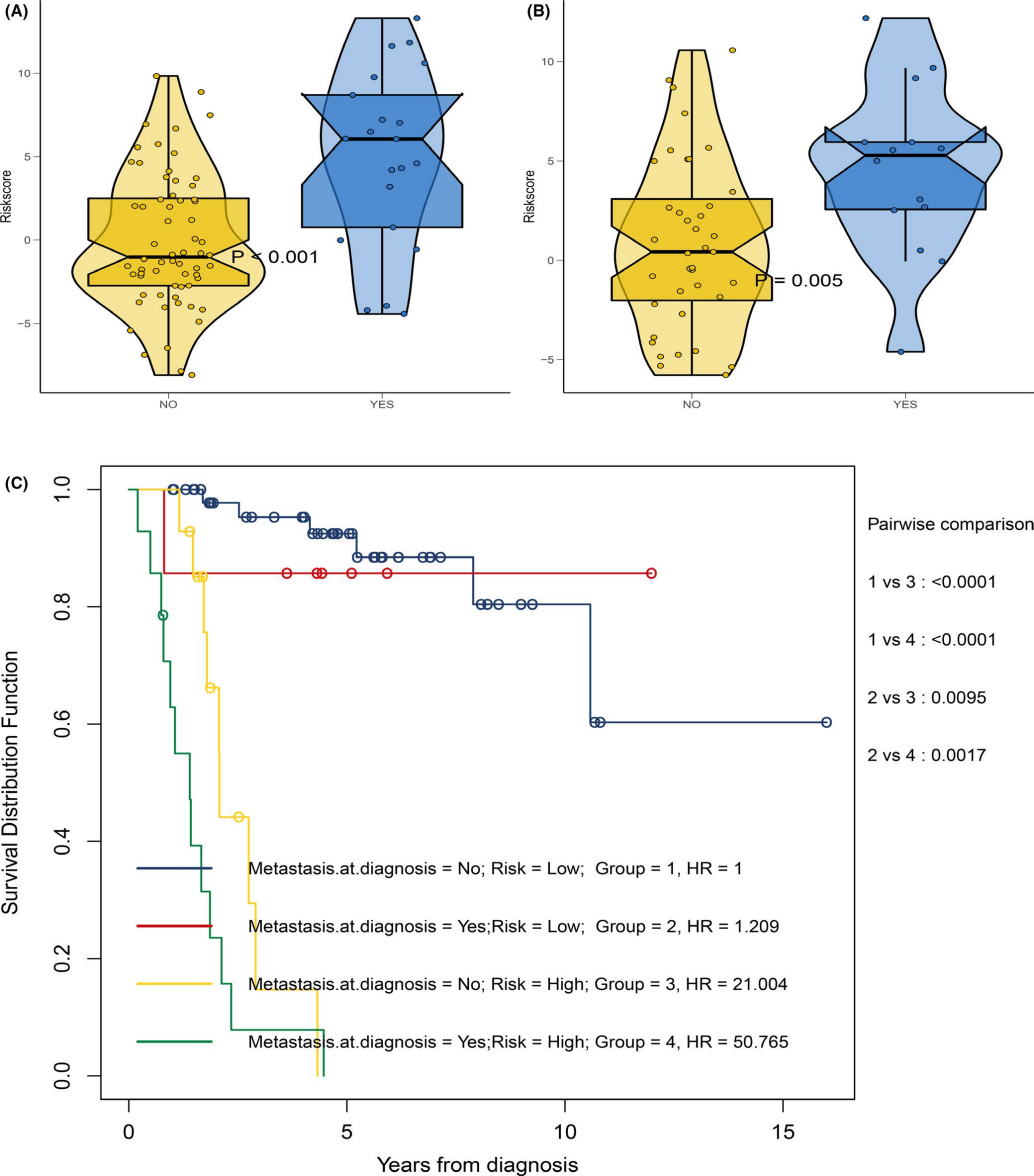
Relationship between MRGP signature and metastasis status. (A) Box violin plot of MRGP values of patients in metastasis group and non‐metastatic group in TCGA cohort. (B) Box violin plot of MRGP values of patients in metastasis group and non‐metastatic group in GSE21257 cohort. (C) Kaplan–Meier survival curves of overall survival for osteosarcoma patients in subgroups stratified by both MRGP groups and metastasis status

Today, the metastatic status remains the primary relevant guideline for the diagnosis and treatment of osteosarcoma patients. Therefore, the relationship between MRGP signature and metastasis status is further explored. As shown in Figure 6AB, in the GSE21257 cohort and TCGA cohort, the MRGP signature value of patients with tumour metastasis is higher. According to the patient's metastatic status and MRGP signature, we divided the osteosarcoma patients in the TCGA cohort into four groups. As shown in Figure 6C, the overall survival among non‐metastatic patients and metastatic patients from the MRGP low‐risk group did not show any significant difference. On the other hand, the MRGP high‐risk group patients from the metastatic group showed a shorter overall survival than the MRGP low‐risk group patients.

**FIGURE 7:**
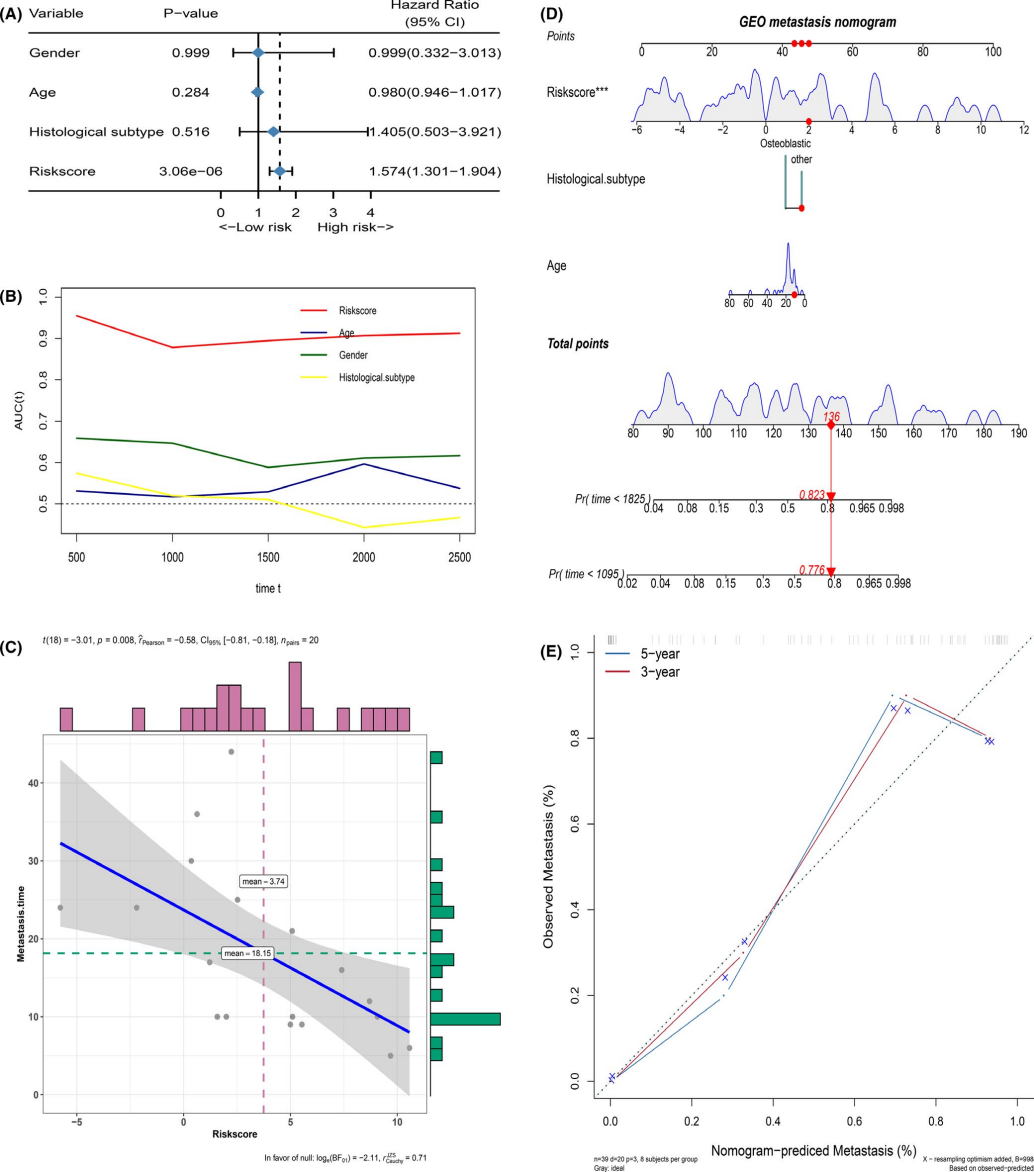
MRGP signature can predict tumour metastasis in patients with osteosarcoma. (A) Forest plots of the associations between various variables and tumour metastasis in the GSE21257 cohort. (B) Time‐dependent ROC curves of tumour metastasis according to MRGP groups in the GSE21257 cohort. (C) Correlation between MRGP value and the time of tumour metastasis. (D) Nomogram to predict the probability of GSE21257 patient's tumour metastasis based on MRGP and clinical variables. (E) Calibration chart in the GSE21257 cohort to verify the accuracy of the nomogram

The GSE21257 dataset includes the patient's metastatic status at the time of diagnosis and whether and when the patient has metastases in the five years following diagnosis. Therefore, we further focus on the GSE21257 dataset. First, we excluded patients with metastases during the diagnosis. Based on the time of sarcoma metastasis, the occurrence of sarcoma metastasis is defined as the outcome. As shown in Figure 7A, patients with high MRGP signatures have a higher risk of metastasis. The results of the multivariate analysis indicate that the MRGP signature is an independent risk factor. Time‐dependent ROC analysis shows that MRGP signatures have better predictive capabilities (Figure 7B). Besides, we further excluded patients without metastasis and also explored the relationship between MRGP signature and the time of metastasis. As shown in Figure 7C, the higher the MRGP signature value is, the earlier the tumour metastasis occurs. Based on the MRGP signature, we constructed a nomogram to predict osteosarcoma patients’ metastasis (Figure 7DE). The nomogram‐C index was 0.849. Unfortunately, only 20 patients are available for analysis, so our results require careful interpretation.

### 3.6 Differences in biological processes and immune infiltrates between MRGP signature group

**FIGURE 8:**
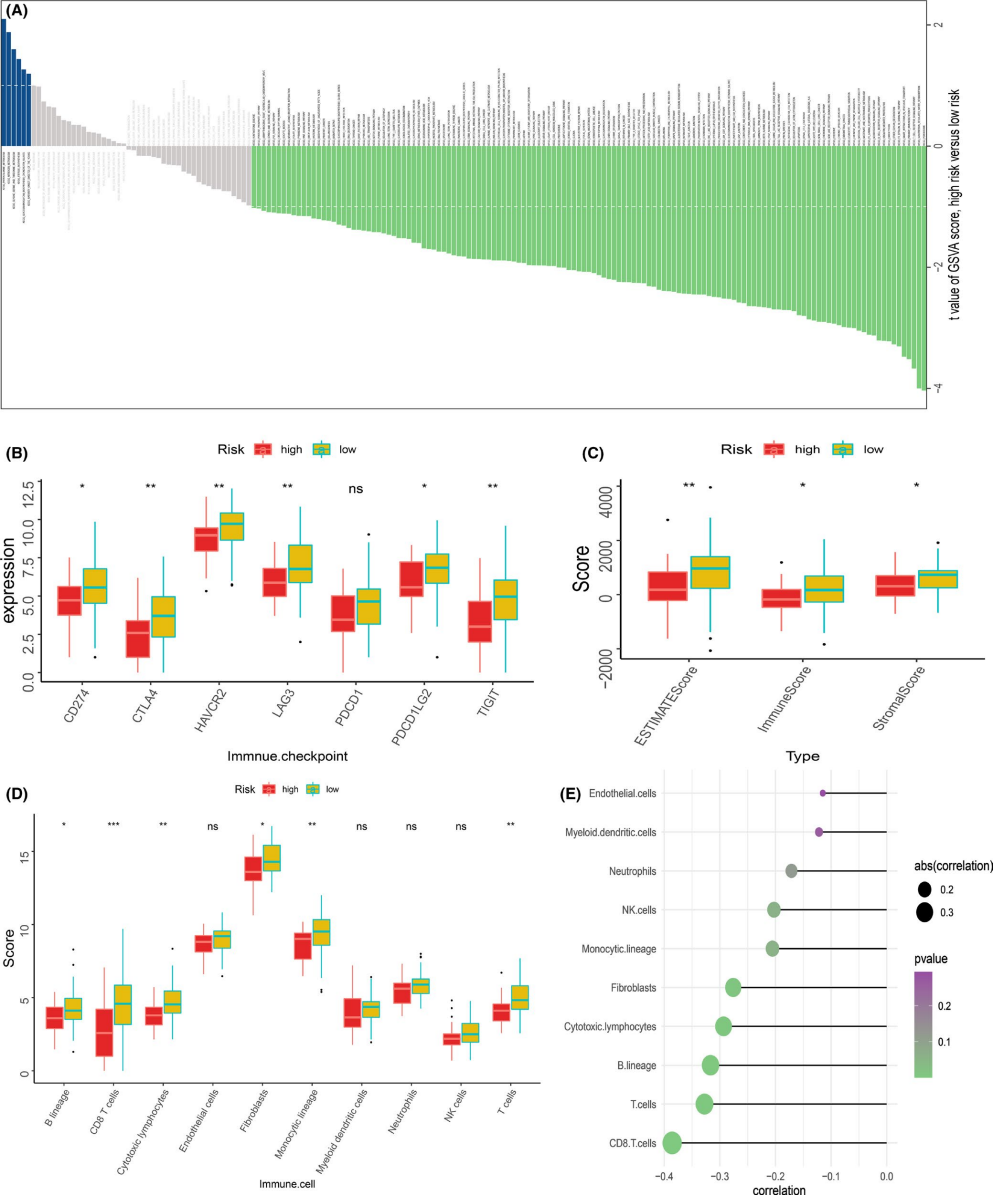
Difference of immune infiltration among patients in MRGP signature group. (A) The bar plot shows the specific metabolism‐associated signatures of the two molecular subtypes. (B) Box plot showing the expression of 7 immune checkpoint genes in two groups of patients. (C) The box plot shows the immune score, stromal score and ESTIMATE score of the two groups of patients. (D) Box plot showing the absolute abundance scores of 8 immune cell and 2 stromal cell populations in two groups of patients. (E) Correlation matrix between absolute abundance scores of immune cells and stromal cells and MRGP values. The size of the bubble represents the degree of correlation, and the colour of the bubble represents the *p*‐value of the correlation. (ns represents no significance, *p < 0.05, **p < 0.01, ***p < 0.001)

The investigation of the differences in biological phenotypes among the MRGP signature groups was performed with the help of GSVA. The biological behaviour of MRGP high‐risk group and MRP low‐risk group patients was significantly different. Table S9 provides detailed results of GSVA. The MRGP high‐risk group patients maintained higher values for metabolic cycles such as phenylalanine metabolism and nitrogen metabolism. In the MRGP low‐risk group, T cell and B cell receptor signalling pathways, peroxisomes, and vasopressin‐regulated water reabsorption pathways are higher (Figure 8A). Since the results of GSVA showed that many immune or inflammation‐related pathways scored higher in patients in MRGP low‐risk group, we further investigated the difference between immune infiltrates in MRGP signature groups. As described in the method, we used the MCP‐counter algorithm to generate absolute abundance scores for ten immune cell and stromal cell populations. The MRGP low‐risk group atients showed a higher abundance of immune‐related cells. Also, the bunch of immune cells has a significant relationship with the MRGP signature value. Furthermore, the immune score, stromal score and estimate score of patients in the MRGP low‐risk group were higher. Finally, we investigated the differences in the expression of seven immune checkpoint genes among the MRGP signature group. Our results show that, except for the PDCD1 gene, all immune checkpoint genes are highly expressed in the MRGP low‐risk group (Figure 8B–E).

### 3.7 Prediction of immunotherapy‐response using MRGP signature

**FIGURE 9:**
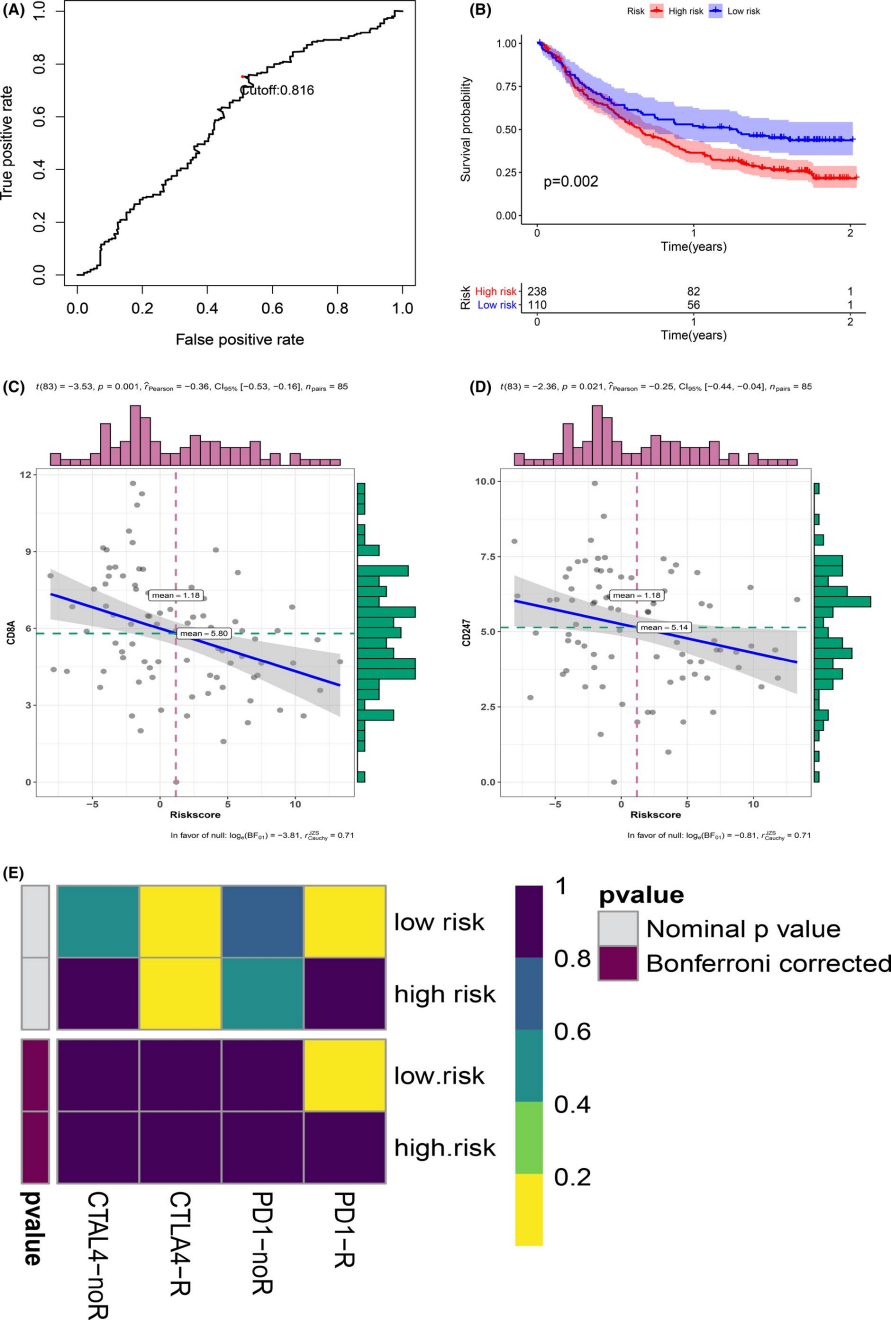
Relationship between MRGP signature and immunotherapy efficiency. (A) ROC curve and cut‐off value according to the overall survival rate of the MRGP group in the IMvigor210 cohort. (B) Kaplan–Meier curves of overall survival according to MRGP groups in the IMvigor210 cohort. (C) Correlation between MRGP value and CD8A gene expression. (D) Correlation between MRGP value and CD247 gene expression. (E) Heatmap of correlation between expression profiles of patients in the MRGP group and patients receiving immunotherapy. The colour of the grid represents the correlation *P*‐value

Due to the significant difference in immune infiltration pattern and immune checkpoint gene expression between the two groups of patients, the relationship between MRGP signature and immunotherapy response was further explored. First, we tested the predictive ability of MRGP signatures in the Imvigor210 data set. Imvigor210 records the expression data of patients with urinary tumours receiving anti‐PD‐L1 immunotherapy. We used MRGP signatures to divide patients into MRGP high‐risk groups and MRGP low‐risk groups. Kaplan–Meier curves show that MRGP high‐risk group patients have a poorer prognosis than MRGP low‐risk group patients (Figure 9AB). Unfortunately, ROC analysis results show that the predictive power of MRGP signatures is limited (AUC = 0.597).

Subsequently, we explored the relationship between MRGP signature and TMIT. Since there is no optimal cut‐off value for TMIT classification, we regard the MRGP signature as a continuous variable. Our results show that the MRGP signature value is negatively correlated with the expression of the CD8A gene and CD247 gene (Figure 9CD). Therefore, the MRGP low‐risk group patients are more prone to be classified as TMIT I.

Finally, the SubMap analysis was used to analyse the relationship between immunotherapy and MRGP signature efficiency. We compared the transcriptome data of patients in MRGP signature groups (MRGP low‐risk group and MRGP high‐risk group) using a published immunotherapy data set with the help of subclass mapping. This dataset contains RNA‐sequence data from 47 melanoma patients undergoing immunotherapy. Our results show a specific association between patients’ expression profiles in the MRGP low‐risk group and patients in the PD‐L1 response group, which indirectly indicates that MRGP low‐risk group patients are more prone to be suitable PD‐L1 therapy. (Figure 9E).

## 4 DISCUSSION

Since 1970, the treatment and prognosis of osteosarcoma have not been changed. Limited understanding of osteosarcoma's complex biological behaviour limits the development of new treatment methods.^6^ The heterogeneity of tumour metabolism might affect patient prognosis and treatment response to a certain extent. Signatures based on metabolic processes have shown a reliable prognostic value in various tumours such as hepatocellular carcinoma and breast cancer.^33^, ^34^ This study used metabolic genes to construct metabolic gene pairs and identified molecular subtypes of osteosarcoma. Research shows significant differences in metabolic characteristics, immune characteristics, and prognosis between the two subtypes. Subsequently, we used Lasso regression to reduce the dimensions to construct MRGP signatures that are expected to be applied clinically. Finally, a signature consisting of 39 metabolic gene pairs was determined. Our signature can effectively identify patients with a high risk of death. It is worth noting that our MRGP is calculated as per the relative ranking of the patient's metabolic gene expression value, without pre‐treatment of the patient's gene expression profile, which allows our signature to overcome the batch effects of the different platforms effectively. Therefore, our signature is more convenient for clinical applications. However, the use of signatures requires verification in an independent data set with complete clinical information.

Disorders of various metabolic pathways, including glucose metabolism and tricarboxylic acid cycle, have been reported in osteosarcoma.^35^, ^36^, ^37^ However, most of these studies focused on studying a single metabolic pathway or a single metabolite. In this study, based on metabolic gene pairs, we comprehensively evaluated the value of metabolic pathways in osteosarcoma. Our results show that metabolic pathways such as transsulfuration, hexosamine biosynthesis and cholesterol biosynthesis in MC2 patients are significantly upregulated and are associated with poor prognosis. Consistent with our findings, a recent study showed that transsulfuration contributes to the de novo synthesis of cysteine in cancer cells. Besides, transsulfuration‐mediated cysteine synthesis is critical for promoting tumour growth in vivo.^38^ The hexosamine biosynthetic pathway is a shunt pathway of glycolysis and is a metabolic node in cancer cells.^39^ Studies have shown that the up‐regulation of the hexosamine biosynthetic pathway is related to tumour survival and chemotherapy resistance.^40^ Inhibiting the biosynthetic pathway of hexosamine can have a significant therapeutic effect on patients with acute myeloid leukaemia.^41^ A recent study showed that targeting endogenous hexosamine biosynthesis makes pancreatic cancer sensitive to anti‐PD1 treatment.^42^ Unfortunately, research on the value of hexosamine biosynthetic in osteosarcoma is very limited.

Similarly, recent studies have shown that intracellular cholesterol can promote tumour formation or growth. There are also reports of cholesterol accumulation in lung cancer, prostate cancer and bone metastases.^43^ Therefore, we have a reason to believe that the differences in the metabolic pathways between the two metabolic clusters lead to different prognoses of the patients. At the same time, our research also provides a possible direction for further studying the value of osteosarcoma metabolic pathways.

Tumour metastasis is one of the leading causes of poor prognosis in osteosarcoma patients.^44^, ^45^ Identifying patients with a high risk of metastasis and enhancing follow‐up of these patients may improve these patients’ prognosis among the complete patient metastasis data in the GSE21257 dataset, we focused on the relationship between MRGP signatures and metastasis in patients with osteosarcoma. Our results indicate that for non‐metastatic patients during the diagnosis, patients in the group with a higher MRGP signature had a significantly increased risk of metastasis within five years. Further analysis showed that patients with high MRGP signature values were more prone to early metastasis. Therefore, it is necessary to strengthen the follow‐up of patients with high MRGP signature value. However, due to sample size limitations, further research is needed to verify our conclusions.

Recent studies on immune checkpoint inhibitor therapy have shown promising clinical benefits.^46^, ^47^, ^48^ As early as 1890, researchers suggested immunotherapy as a related treatment strategy for sarcoma.^49^ Unfortunately, the results of several recent clinical trials investigating the value of immune checkpoint inhibitors in osteosarcoma have been disheartening.^50^, ^51^, ^52^ Identifying suitable patients is essential to benefit from this treatment strategy. Hence, we used three methods to evaluate the relationship between MRGP signature and immunotherapy efficiency. First, we found that the MRGP signature can predict the outcome after anti‐PDL1 in mUC. Unfortunately, the predictive power of MRGP signatures is limited, which perhaps due to the heterogeneity between tumours. Subsequently, we further explored the relationship between MRGP signature and TMIT. Similarly, patients with low MRGP signature values are more prone to be classified as TIMT I due to higher CD8A gene expression and higher CD274 gene expression. This means that the MRGP low‐risk group may be suitable for anti‐PD‐1 therapy. Finally, the results of SubMap analysis also revealed that the expression profile of MRGP low‐risk group patients is associated with the expression profile of patients responding to PD‐L1 treatment. In conclusion, we speculate that MRGP low‐risk group patients are more likely to be suitable for anti‐PD‐L1 therapy. However, further clinical trials are needed to verify these conclusions.

Nowadays, many signatures based on certain characteristics have been developed to predict the prognosis of tumour patients.^53^, ^54^ Recent studies have constructed several signatures that can predict the overall survival of osteosarcoma.^55^, ^56^, ^57^ Compared with previous studies, our study incorporates the most comprehensive data set and achieved more consistent results, enhancing the persuasiveness of our study. Also, compared with previous studies, our signature has more powerful predictive power in predicting the overall survival of osteosarcoma. In addition, because our signature is calculated based on the relative ranking of the patient's metabolic gene expression value, it can effectively overcome the batch effect between different platforms and has greater clinical application potential. Finally, this is the first comprehensive evaluation of the value of metabolic genes in osteosarcoma as far as we know.

Limitations of our study must be stated. First, some of the data sets included in the study have small sample sizes, with only 53 patients in GSE21257 and 37 patients in GSE39055, respectively. However, to the best of our knowledge, only these three data sets have both patient transcriptome data and comprehensive clinical information. Secondly, this study is a retrospective study, and there is some heterogeneity in the patient population. Finally, because of insufficient data on immunotherapy in osteosarcoma patients, it is impossible to directly explore the connection between MRGP signature and immunotherapy efficiency in patients with osteosarcoma.

## 5 CONCLUSIONS

In conclusion, our study divided osteosarcoma patients into two clusters from the perspective of metabolism. Among them, metabolic cluster 1 is related to metabolic characteristics such as thromboxane biosynthesis, primary bile acid biosynthesis, glycosaminoglycan degradation and other glycan degradation and has better overall survival. Metabolic cluster 2 patients are related to metabolic characteristics such as steroid biosynthesis, cholesterol biosynthesis, hexosamine biosynthesis, transsulfuration and have a poor prognosis. The subsequently developed signature consisting of 39 metabolic gene pairs can well predict the overall survival and metastasis of patients with osteosarcoma. Finally, the IMvigor data set, TMIT and SubMap analysis results indicate that our signature may help identify patients with osteosarcoma suitable for immunotherapy.

## ETHICAL APPROVAL STATEMENT

The TCGA and GEO database is publicly available, and our study was performed based on the guideline of these databases. All patient information was anonymised and de‐identified in the TCGA and GEO database. Thus, our study was exempted from the ethics committee approval and patients’ informed consent.

## CONFLICT OF INTEREST

The authors confirm that there are no conflicts of interest.

## AUTHOR CONTRIBUTION

L‐LQ collected and analysed the data and wrote the paper. Z‐LH and Y‐YB assisted in collecting the data and participated in the writing. L‐XC, Z‐Y, Manhas, W‐J, L‐T, and L‐YK assisted in the design of this study. L‐JZ and Z‐Y are responsible for all the integrity of data and the accuracy of data analysis. All authors have thoroughly revised the manuscript.

## Supporting information

Fig S1

Fig S2

Table S1‐S9

## Data Availability

RNA‐seq data of the TCGA cohort can be obtained from the TCGA database (https://portal.gdc.cancer.gov). Clinical data of these patients can be obtained from the TARGET database (https://ocg.cancer.gov/programs/target). GSE21257 (http://www.ncbi.nlm.nih.gov/geo/query/acc.cgi?acc=GSE21257). GSE39055 (http://www.ncbi.nlm.nih.gov/geo/query/acc.cgi?acc=GSE39055). IMvigor210 R package ‘IMvigor210’.
