# Supplementary figures and images for: Signature based on metabolic‐related gene pairs can predict overall survival of osteosarcoma patients
Source: Cancer Med. 2021 May 28;10(13):4493–509. doi: 10.1002/cam4.3984 (PMC8267140; doi:10.1002/cam4.3984)

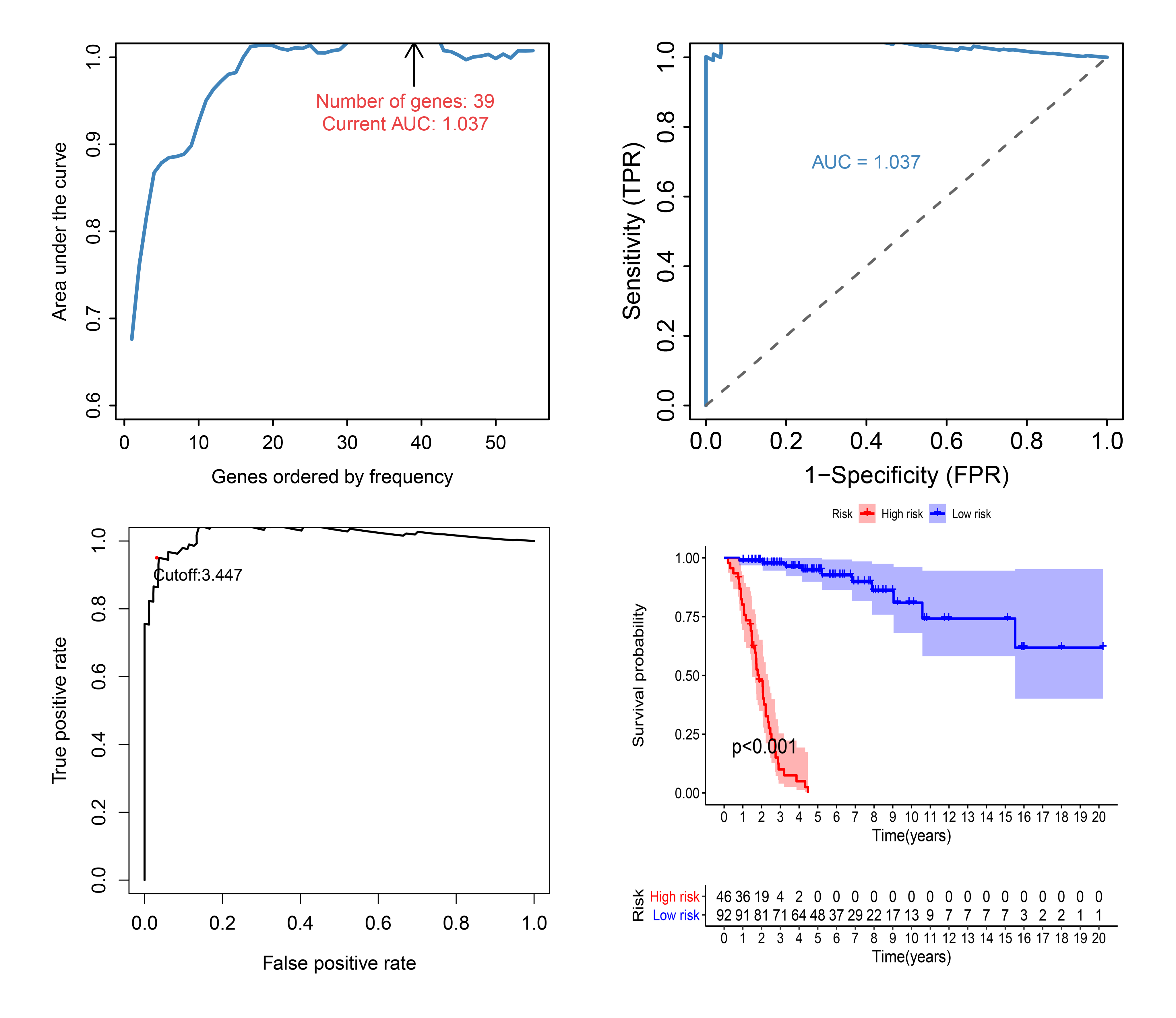

Supplement: Supplementary file 1 — Fig S1 [file CAM4-10-4493-s001.tif]

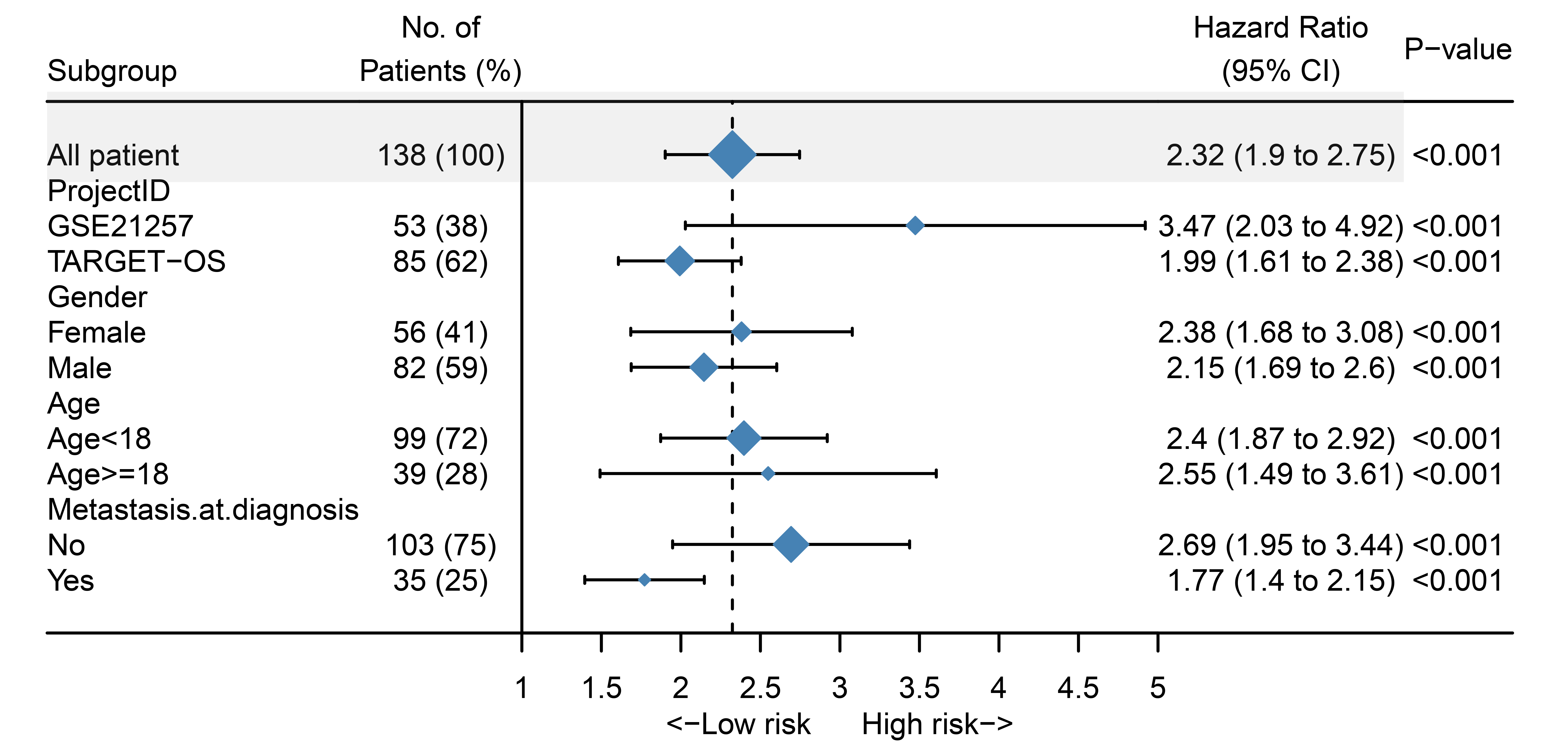

Supplement: Supplementary file 2 — Fig S2 [file CAM4-10-4493-s003.tif]
